# A Microstirring Pill Enhances Bioavailability of Orally Administered Drugs

**DOI:** 10.1002/advs.202100389

**Published:** 2021-05-18

**Authors:** Rodolfo Mundaca‐Uribe, Emil Karshalev, Berta Esteban‐Fernández de Ávila, Xiaoli Wei, Bryan Nguyen, Irene Litvan, Ronnie H. Fang, Liangfang Zhang, Joseph Wang

**Affiliations:** ^1^ Department of Nanoengineering and Chemical Engineering Program University of California San Diego La Jolla CA 92093 USA; ^2^ Department of Neurosciences University of California San Diego La Jolla CA 92093 USA

**Keywords:** active drug delivery, bioavailability, microstirring pills, porcine models, translational medicine

## Abstract

Majority of drugs are administered orally, yet their efficient absorption is often difficult to achieve, with a low dose fraction reaching the blood compartment. Here, a microstirring pill technology is reported with built‐in mixing capability for oral drug delivery that greatly enhances bioavailability of its therapeutic payload. Embedding microscopic stirrers into a pill matrix enables faster disintegration and dissolution, leading to improved release profiles of three widely used model drugs, aspirin, levodopa, and acetaminophen, without compromising their loading. Unlike recently developed drug‐carrying nanomotors, drug molecules are not associated with the microstirrers, and hence there is no limitation on the loading capacity. These embedded microstirrers are fabricated through the asymmetric coating of titanium dioxide thin film onto magnesium microparticles. In vitro tests illustrate that the embedded microstirrers lead to substantial enhancement of local fluid transport. In vivo studies using murine and porcine models demonstrate that the localized stirring capability of microstirrers leads to enhanced bioavailability of drug payloads. Such improvements are of considerable importance in clinical scenarios where fast absorption and high bioavailability of therapeutics are critical. The encouraging results obtained in porcine model suggest that the microstirring pill technology has translational potential and can be developed toward practical biomedical applications.

## Graphical abstract

Microstirring pill platform technology offers a built‐in mixing capability for in vivo oral drug delivery and leads to significantly enhanced drug absorption and bioavailability. In vivo studies using murine and porcine models demonstrate that the localized stirring capability of microstirrers results in enhanced bioavailability of drug payloads. This elegant technology offers considerable promise for oral delivery of therapeutics.

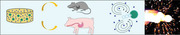

## 1 Introduction

Oral drug formulations are widely used due to ease of administration, high patient compliance and safety, and cost‐effective manufacturing.^[^ ^1^ ^]^ Nevertheless, the oral delivery route has some inherent disadvantages when compared with other methods of administration, including reduced control over the drug release rate, limited target specificity and absorption across the mucosal barrier, drug degradation in the gastrointestinal (GI) tract, and side effects due to the high dose required for achieving the desired therapeutic effect.^[^ ^2^, ^3^ ^]^ One of the main parameters to assess drug performance is bioavailability, which is defined as the fraction of dosed drug that reaches systemic circulation. Achieving high bioavailability depends strongly on the drug solubility, GI absorption, and permeability, and often involves high dosing and undesired side effects.^[^ ^1^ ^]^ Thus, extensive efforts have been dedicated to enhancing drug bioavailability not only by modifying drug molecules themselves but also by developing formulation systems. For instance, nanotechnology has been utilized to increase bioavailability of certain drugs. One approach encapsulated insulin inside polymeric nanoparticles for sustained delivery through oral administration, with the size and large surface area of nanoparticles leading to improved absorption and the therapeutic index.^[^ ^4^, ^5^ ^]^

Pills are solid drug formulations, comprised of functional active pharmaceutical ingredients and inactive excipients in powder, crystalline, or granular forms, that are commonly manufactured by compression techniques.^[^ ^6^ ^]^ Once a pill is ingested, it disintegrates in the stomach upon contacting GI fluids and releases its drug payload, followed by drug absorption in either the stomach or intestine.^[^ ^7^ ^]^ The pill disintegration, drug dissolution, and dispersion processes, and GI permeability, have profound impacts on the degree of drug absorption and on the bioavailability of the therapeutic payload.^[^ ^8^, ^9^ ^]^ Several pharmaceutic strategies have been developed in pills to improve the drug bioavailability, such as a pullulan‐based pill loaded with rosuvastatin flexible chitosomes,^[^ ^10^ ^]^ a cefdinir‐cyclodextrin complex in tablets for improved drug dissolution rate,^[^ ^11^ ^]^ and orodispersible tablets.^[^ ^12^ ^]^ In addition, effervescent pills, which generally contain a mixture of acid salts and carbonate ion salts that release carbon dioxide upon contact with water, can facilitate faster absorption. Moreover, “smart pills/capsules” have been introduced recently, including a luminal unfolding microneedle injector pill for insulin delivery,^[^ ^13^ ^]^ a capsule for oral once‐weekly drug delivery system for human immunodeficiency virus (HIV) treatment,^[^ ^14^ ^]^ and a capsule for monitoring GI health.^[^ ^15^ ^]^

Here we show a unique microstirring pill platform technology with built‐in in situ stirring capability for oral drug delivery with enhanced drug uptake and bioavailability. We hypothesize that the inclusion of chemically‐powered microstirrers into a pharmaceutical pill will enhance the drug dissolution and dispersion in the stomach fluid, leading to faster absorption and increased bioavailability. To test our hypothesis, we use magnesium (Mg)‐based Janus microparticles (often called “microengines”) with self‐propulsion ability to fabricate microstirrers. Such microstirrers consist of 25‐µm Mg microparticles, partially coated with a thin titanium dioxide (TiO_2_) layer, where the fabrication process leaves a small opening for microbubbles to exit and propel the microparticle upon reacting with appropriate chemical fuel.^[^ ^16^ ^]^ Furthermore, Mg is an excellent candidate for use in the body as there is high tolerance for ionic Mg in vivo and excess Mg can be easily removed or absorbed.^[^ ^17^ ^]^ Major developments in the field of synthetic microengines over the past decade have led to important advances that can benefit medicine.^[^ ^18^, ^19^, ^20^, ^21^, ^22^ ^]^ Synthetic microengines, capable of converting energy into mechanical motion, can be powered by different sources including chemical, magnetic, electric, and optical, among others.^[^ ^22^ ^]^ Chemically powered microengines have gained particular attention for in vivo biomedical applications, mainly due to their autonomous self‐propulsion, and they have shown benefits for enhanced delivery of therapeutic cargoes with deeper tissue retention.^[^ ^23^, ^24^, ^25^, ^26^, ^27^ ^]^

These Mg microstirrers are incorporated as an excipient into a solid lactose/maltose pill. Note that in the microstirrer pill formulation the therapeutic drugs and the microstirrers are decoupled, which allows for high drug loading while providing efficient stirring action that enhances the drug release, distribution, and absorption once the pill reaches the stomach. A series of in vitro characterizations demonstrate the effective mixing capability of the Mg microstirrers under static and dynamic conditions, along with their ability to enhance local fluid transport. In vivo studies using a murine animal model demonstrate that the in situ stirring capability of Mg microstirrers offers enhanced absorption and bioavailability, and this leads to a faster elevation of serum drug levels. Experimental demonstration and verification of the microstirrer pill in a porcine model, which is physiologically closer to humans, further highlight the translational potential of this technology.

Overall, these findings show that by co‐encapsulating Mg microstirrers as an excipient to a pill formulation along with therapeutic drugs, the microstirrer pill can effectively modulate and enhance the bioavailability of common orally delivered drugs both immediately post administration and at longer time scales. Unlike previous studies where synthetic microengines were loaded with therapeutic agents to perform active delivery, in this platform Mg microstirrers are not associated with the payloads, allowing for a better drug distribution and absorption; moreover, there is no more limitation in the payload loading than the pill capacity, and the fabrication process is simpler compared to older systems. As the use of microstirrers is independent of the loaded drugs, such microstirrer pill can be a platform technology broadly applicable for numerous types of oral drugs.

## 2 Results and Discussion

### 2.1 Microstirring Pill Concept and In Vitro Self‐Stirring Capabilities

**Figure 1:**
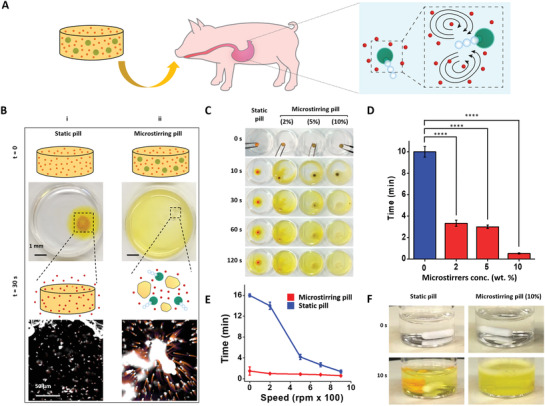
In vitro dissolution rate and self‐stirring capability of microstirring pills. A) Schematic illustration of the self‐stirring and mixing capability of a microstirring pill after in vivo administration. B) Schematics and i) images of static and ii) microstirring pills, demonstrating the faster dissolution rate of microstirring pills and their improved payload dispersion. C) Time‐lapse images showing the dissolution of a static pill and microstirring pills (prepared with 2%, 5%, and 10% of microstirrers, by mass) in 2 mL of 0.7 m HCl solution (corresponding to Video S1, Supporting Information). D) Comparison of dissolution times of static and microstirring pills (prepared with different microstirrer loadings) in 0.7 m HCl solution. One‐way ANOVA, ****p < 0.0001. E) Comparison of dissolution times of static and microstirring pills (prepared with 10% microstirrers) in 0.7 m HCl solution under stirring conditions (0 to 900 rpm). F) Time‐lapse images displaying the dissolution of a static pill and a microstirring pill (10% microstirrers) after 10 s in a stirred 0.7 HCl solution at 200 rpm (corresponding to Video S2, Supporting Information).

**Figure** **1**A schematically illustrates the overall microstirrer pill concept, in which Mg‐based microstirrers (Figure S1, Supporting Information) react in acidic gastric conditions to generate gas microbubbles, thus inducing a stirring effect which leads to significantly faster pill dissolution and rapid dispersion of the drug payload. In addition to the microstirrers and drugs, the pill consists of a matrix formed from a biocompatible combination of lactose and maltose. To evaluate and demonstrate the capabilities of the microstirring pill strategy, we selected three model drugs, aspirin (ASA; acetylsalicylic acid), levodopa (L‐Dopa), and acetaminophen (APAP). Figure 1B illustrates the overall ability of the Mg microstirrers to induce self‐stirring in solution that leads to a faster pill dissolution rate when compared to static pills (traditional pills without microstirrers in their composition). The schematic illustrations and corresponding images show the dissolution process of a static pill and a microstirring pill in 0.7 m hydrochloric acid (HCl) solution. The optical images taken after 30 s of immersion in the HCl solution clearly demonstrated the rapid microstirring pill dissolution, as indicated from the uniform diffusion of the yellow dye. On the contrary, static pills dissolved significantly slower, with the dye spreading dominated by passive diffusion. The microscopy images show the pill formulations with tracer particles, which were used to visualize the fluid mixing effect exerted by the encapsulated Mg microstirrers.

To gain insights into the effect of such self‐stirring on pill disintegration and dissolution, dye‐loaded pills with different microstirrer loadings (2, 5, and 10 wt%) were prepared and further tested in 0.7 m HCl (Figure 1C and Video S1, Supporting Information). Time‐lapse photographic imaging was used to visualize the dissolution process and corresponding dye diffusion at different times ranging from 0 to 120 s. As displayed by the images, just 10 s post‐immersion in HCl solution, the yellow dye coming from microstirring pills permeated a significant portion of the petri dish volume, whereas the dye coming from the static pill was restricted to the pill perimeter. Another difference observed when using microstirring pills was the presence of gas microbubbles, reflecting the efficient reaction of the Mg microstirrers within the acidic solution. As expected, the pill dissolution process was dependent on the loading of the microstirrers and time, as indicated from the even distribution of the yellow dye in the images taken at 120 s. Pills containing 2%, 5%, and 10% microstirrers were dissolved 3.0, 3.3, and ten times faster than static pills, respectively (Figure 1D).

Aiming at mimicking the dissolution of the microstirring pill under the natural movement of the gastric environment, the microstirring pill dissolution was further evaluated under dynamic conditions. For this study, the dissolution time of static pills and microstirring pills (prepared with 10% microstirrers) were compared at different external fluid stirring speeds ranging from 0 to 900 rpm (Figure 1E). The microstirring pill formulation exhibited a dissolution profile that was significantly faster than that of the static pill in general. As expected, the gap between the two pills decreased at higher fluid stirring speed values. Notably, the 10.7‐fold faster pill dissolution time when working at 200 rpm, which is a speed that simulates the fluid hydrodynamics exerted on hydrophilic tablets within the GI,^[^ ^28^ ^]^ suggested that the microstirring pills could induce faster pill dissolution in an in vivo setting.^[^ ^6^, ^28^ ^]^ Time‐lapse imaging further illustrated the different dissolution rates of static and microstirring pills after 10 s in 0.7 m HCl and at 200 rpm (Figure 1F; Figure S2 and Video S2, Supporting Information). While the microstirring pill was almost completely dissolved, the static pill maintained most of its structure after the same period. Overall, these in vitro findings demonstrate that microstirring pills offer faster dissolution profiles with enhanced payload dispersion when compared to the corresponding static pills.

### 2.2 Self‐Stirring Effect of Microstirrers on Tracer Particles and Drug Payloads

**Figure 2:**
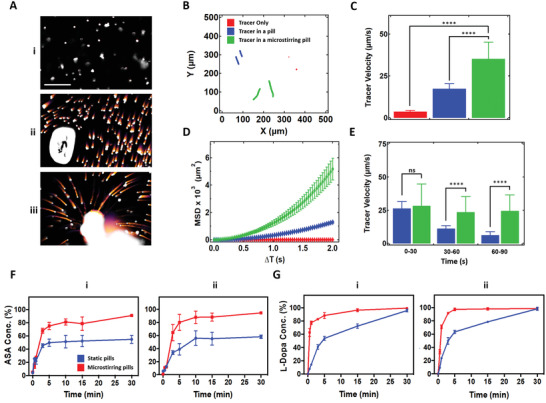
Self‐stirring effect of microstirrers on tracer particles and drug payloads. A) Visualization of fluid mixing generated by overlapping a stack of 30 color‐inverted bright‐field images corresponding to 1 s of motion. i) Tracer particles alone in gastric fluid, ii) tracer particles loaded into a static pill in gastric fluid, and iii) tracer particles loaded into a microstirring pill in gastric fluid were studied (corresponding to Video S3, Supporting Information). Scale bar: 50 µm. B) Optical trajectories corresponding to (a). C) Velocity of tracer particles over a representative 2 s duration from the midpoint of the microstirrer lifetimes. One‐way ANOVA, ****p < 0.0001. D) Mean squared displacement (MSD) of tracer particles alone in solution, released from a static pill, and released from a microstirring pill from the same representative 2 s duration in Figure 2C. E) Velocity of tracer particles for representative 30 s durations over the microstirrer lifetimes. One‐way ANOVA, ****p < 0.0001; ns = not significant. F) Comparison of in vitro dissolution profiles of aspirin (ASA) between static and microstirring pills made with i) laboratory prepared excipients ii) and commercial excipients. G) Comparison of in vitro dissolution profiles of levodopa (L‐Dopa) between static and microstirring pills made with i) laboratory prepared excipients and ii) commercial excipients.

To further elucidate the role of the microstirrers in shortening the pill dissolution times, tracer particles were employed to extract important mixing parameters. Polystyrene tracer particles, 2 µm in size, were loaded into static and microstirring pills, and their positions were tracked over time. To illustrate the differences in tracer particle motion, 30 sequential images were stitched together from a video capture of a dissolution event (**Figure** **2**A and Video S3, Supporting Information). Tracer particles alone in dissolution media only experienced Brownian motion and thus did not exhibit “tails.” For the static pill, short tails were visualized, which resulted from the convective flows associated with pill dissolution. The tails displayed by tracers released from the microstirring pill were significantly longer due to the increased convective flow associated with the microstirrers. This was also reflected when tracking individual tracers over a representative 2 s duration taken from the midpoint of a microstirrer's lifetime, where it could be seen that those released from the microstirring pill had substantially larger displacement (Figure 2B). In terms of average velocity, there was also a ≈ten times difference between tracers released from a microstirring pill versus controls placed directly into dissolution media. To further describe these differences, we calculated the mean square displacement (MSD) versus delay time (Δ*T*) for each of the three scenarios from the representative 2 s durations in Figure 2C. The linear nature of the control tracers in simulated gastric fluid confirmed their Brownian motion behavior. The MSD of the tracers in a static pill and microstirring pill exhibited a parabolic trend with MSD ≈ Δ*T* ^2^ (Figure 2D). This superdiffusive behavior suggested departure from Brownian motion and enhanced transport at the microscale. To assess the self‐stirring capability of the microstirrers, we extracted effective diffusion coefficients for the tracer particles, which were calculated based on Equation (1).

(1) $$\mathrm{MSD}\left(\Delta T\right)=2\times d\times D_{\mathrm{eff}}\times \Delta T$$

Where *d* is the dimensionality of the system (in this case *d* = 2) and *D*_eff_ is the effective diffusion coefficient. Using the maximum slope of the curves,^[^ ^29^ ^]^ we estimated that tracers experiencing purely Brownian motion had a *D*_eff_ value of 0.74 µm^2^ s^–1^. Tracers released from the static pill had a larger *D*_eff_ value of 279 µm^2^ s^–1^ while tracers being actively stirred by microstirrers exhibited a *D*_eff_ value of 1197 µm^2^ s^–1^. Such significant differences in *D*_eff_ clearly illustrate the enhanced motion of the tracers associated with the convective flows of pill dissolution, with the effect being more pronounced in the case of the microstirring pill (Figure S3, Supporting Information). To gain further insights into the overall performance of the microstirrers over their whole lifetime, we analyzed tracer motion at different time points during the pill dissolution (Figure 2E). We observed that the speed of the tracers released from a static pill decreased with time, confirming that the convective flows moving the tracers were mainly due to the dissolution of the pill. On the other hand, the velocity of the tracers released from the microstirring pill remained relatively constant and was consistently higher than those from a static pill in all three 30 s intervals during the lifetime of the microstirrers. This observation suggested that microstirrers contribute to enhanced fluid mixing not only on short time scales (representative 2 s durations from the midpoint of their lifetime, Figure 2C) but also over the entire pill dissolution timeframe (representative 30 s durations over the microstirrer lifetime, Figure 2E).

Subsequently, the microstirrer self‐stirring effect was evaluated by assessing the dissolution profiles of various drugs. In this case, we studied ASA, L‐Dopa, and APAP, three widely used medicines for antiplatelet therapy, Parkinson's disease treatment, and as an analgesic/antipyretic, respectively, where fast drug absorption is essential. To carry out this study, microstirrers were incorporated along with ASA, L‐Dopa, or APAP into pills, and the dissolution profiles were compared to the ones obtained from static pills loaded with the same drugs. For our laboratory preparation, a pill matrix consisting of only maltose and lactose was used. In order to evaluate the self‐stirring effect in commercial‐type formulations, separate pills were also prepared by triturating ASA, Sinemet, and Tylenol pills, incorporating microstirrers into the mixture, and then compressing all excipients to fabricate new pills. The same procedure was followed for static commercial pills without adding microstirrers. Each of the prepared pills was dissolved in 10 mL of gastric fluid simulant at 37 °C. Then, aliquots were taken at different times during an interval of 30 min to quantify the concentration of ASA by ELISA, or L‐Dopa and APAP by square wave voltammetry (Figure 2F,G, and Figure S4, Supporting Information). A faster dissolution was achieved when microstirrers were in the pill formulations, demonstrating higher drug release at each time point for all scenarios. For ASA, the profiles for microstirring laboratory and commercial‐type pills were similar in shape, and ≈90% of the drug was dissolved after 30 min, which was ≈1.6‐fold higher than that for the static pills. A slightly different behavior was observed for the pills loaded with L‐Dopa. Whereas full drug dissolution was not observed for static pills loaded with ASA, full release was achieved for static L‐Dopa pills, albeit the kinetics were significantly delayed compared to their microstirrer counterparts. Such behavior likely reflects the higher solubility of L‐Dopa in water compared to ASA (66 and 3 mg mL^–1^, respectively). The same trends were obtained when APAP microstirring pills were tested. Overall, the inclusion of microstirrers to the pill formulations enabled a faster release of the drug, which could be crucial in certain emergency medical applications.

### 2.3 In Vivo Evaluation of Microstirring Pills in a Murine Model

**Figure 3:**
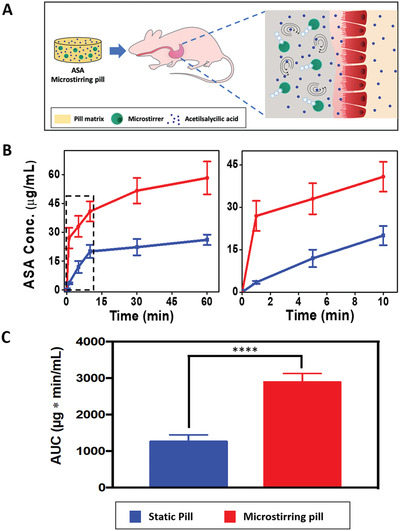
In vivo ASA delivery using microstirring pill in a murine model. A) Schematic showing the concept for in vivo ASA absorption kinetic study using static and microstirring pills (ASA, 0.6 mg). B) Serum concentration of ASA after administration of static pills and microstirring pills (*n* = 6). Left: complete kinetic profiles over 60 min; right: kinetic profiles over the initial 10 min. C) ASA AUC values for static and microstirring pills over 60 min. Unpaired Student's *t*‐test, ****p < 0.0001.

With the microstirring pills providing faster release of ASA, L‐Dopa, and APAP, we performed a suite of in vivo studies using murine and porcine models. The goal of these animal studies was to evaluate whether the self‐stirring effect could help to accelerate the absorption of orally delivered drugs, and, as a consequence, offer higher bioavailability and faster pharmacokinetic uptake profiles. ASA was chosen as the model drug for these in vivo studies because it is absorbed both from the stomach and from the upper intestinal tract.^[^ ^30^ ^]^ For the murine study, 1 × 3 mm microstirring pills were prepared, and their disintegration and dissolution capabilities were tested, as shown in Figure S5, Supporting Information. **Figure** **3**A illustrates the concept of the in vivo study performed using the murine model. Mice (*n* = 6) were administered with static or microstirring pills, both containing 0.6 mg ASA. Then, blood samples were collected at 1, 5, 10, 30, and 60 min post‐administration in order to quantify the ASA concentrations (Figure 3B). From the first time point, a significant difference between the serum drug concentrations was observed, with the value being ≈8.0‐fold higher when microstirring pills were administered. The improved drug bioavailability persisted over the entire monitoring period, with the final serum ASA levels plateauing at greater than double the concentration achieved with static pills. The amount of absorbed ASA (Figure S6, Supporting Information) was calculated on the basis of a mouse weight of ≈20 g and the corresponding circulating blood volume of ≈2.4 mL. The absorbed fraction obtained with microstirring pills 60 min post‐administration was ≈2.2‐fold higher than the one obtained with static pills (23.4% and 10.4% of the administered dose, respectively), reflecting a greater fraction of the administered dose that reached the circulation using the microstirring pills. A significantly larger difference in absorbed fraction (≈8‐fold) is observed 1 min after the drug administration (Figure S6), with 10.8% and 1.4% of administrating the microstirring and static pills, respectively. Such behavior is particularly important in emergency medical situations, when high blood drug concentrations are needed immediately after the pill ingestion. Specifically, due to its antiplatelet properties, ASA is highly recommended for immediate treatment of patients suspected of having a heart attack.^[^ ^31^ ^]^ Figure 3C displays the area under the curve (AUC) values obtained with static and microstirring pills at 60 min post‐administration. The AUC obtained with ASA microstirring pills was ≈2.4‐fold higher than with ASA pills (2890 versus 1260 µg min mL^–1^, respectively), reflecting a larger dose fraction of the drug reaching the systemic circulation over this period of time using microstirring pills. These findings here clearly demonstrate that the use of microstirrers as an excipient in a pill formulation greatly enhances drug absorption and bioavailability, resulting in both accelerated and elevated serum concentrations.

### 2.4 In Vivo Evaluation of Microstirring Pills in a Porcine Model

**Figure 4:**
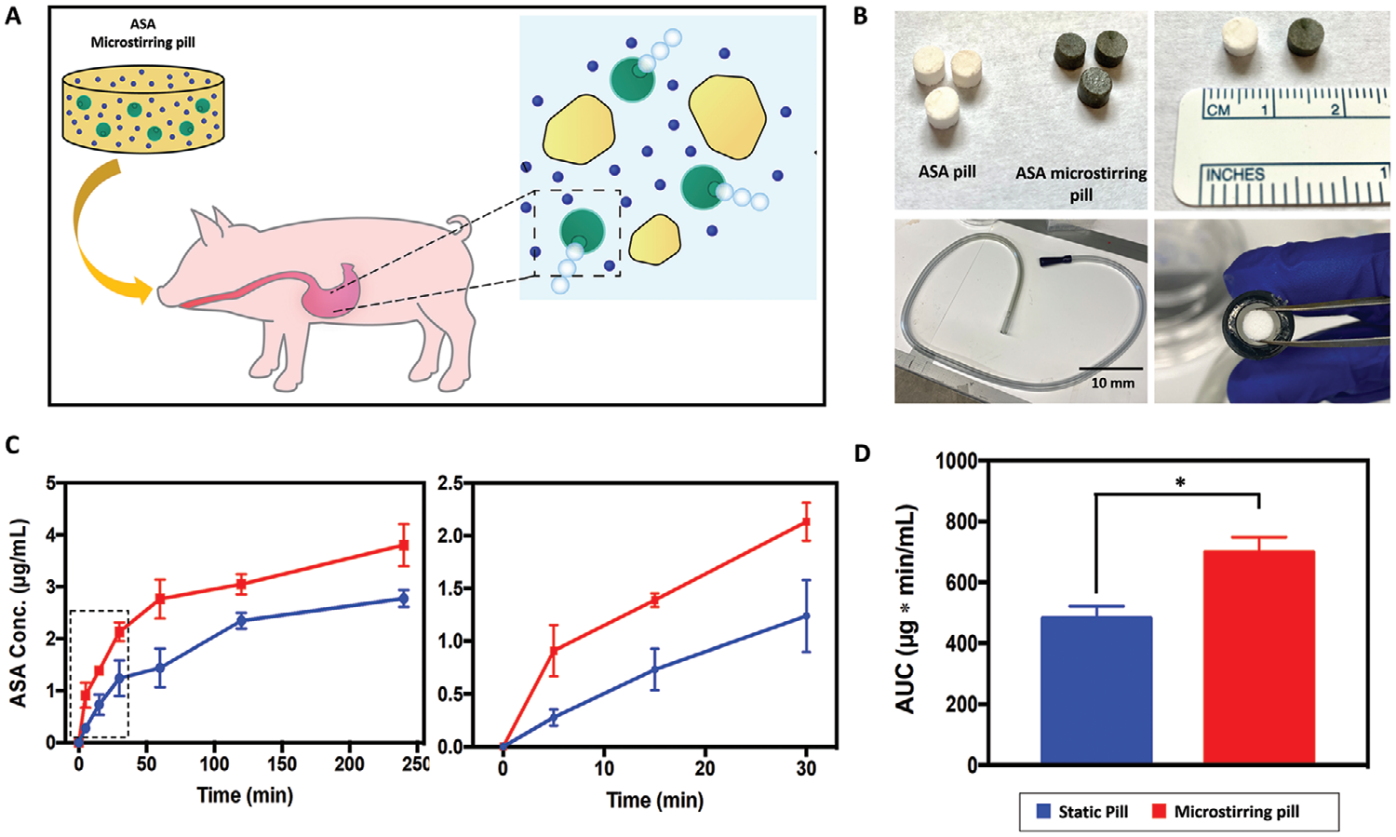
In vivo ASA delivery using microstirring pill in a porcine model. A) Schematic of microstirring pill technology and its application in a porcine model. B) Images of ASA‐loaded static and microstirring pills (top row) and the tube used to perform the pill administration by oral gavage (bottom row). C) Serum concentration of ASA after administration of ASA‐loaded microstirring pills and static pills (*n* = 3). Left: complete kinetic profile over 250 min; right: kinetic profile over the initial 30 min. D) ASA AUC values for static and microstirring pills over 4 h. Unpaired Student's *t*‐test, *p < 0.05.

Based on the encouraging results in the murine model, the ASA absorption in vivo study was extended to a porcine model. As a larger animal, pigs display more similarity to humans with regards to the GI tract and are commonly used for predicting human bioavailability of orally administered drugs.^[^ ^32^ ^]^ The general concept of this experiment is illustrated in **Figure** **4**A, in which a microstirring ASA pill is administered to a pig; when the pill reaches the stomach, it starts to dissolve while the microstirrers are activated, allowing for a faster dissolution and a greater drug dispersion due to the in situ self‐stirring effect. Pills were fabricated by forming in a mortar a paste composed of ASA, microstirrers, and the pharmaceutical excipients lactose and maltose, followed by a hardening process within an appropriately sized mold (Figure S7, Supporting Information). The resulting 3 × 5 mm pills were designed to fit in the oral gavage feeding tube that was used for the pill administration (Figure 4B). When looking at the kinetic profiles obtained from this study (Figure 4C), a similar trend was observed compared with those of the mouse study. The ASA blood concentration at the first 5 min time point was 3.26 times higher when microstirrers were included inside the pill, and it remained ≈1.90 and 1.72‐fold higher at 15 and 30 min post‐administration, respectively. After 4 h of the administration, the ASA concentration obtained with microstirring pills was ≈1.4 times higher. Figure 4D displays the AUC values obtained with static and microstirring pills during this study. The AUC obtained with ASA microstirring pills was ≈1.5‐fold higher than with ASA pills (698.9 versus 481.5 µg min mL^–1^), indicating that a higher fraction of the drug was absorbed and reached blood circulation over 4 h with microstirring pills. Similar to the murine model, the bloodstream ASA values do not reach the same value at the end of the study as the active pills offer enhanced bioavailability and absorption. These encouraging results, obtained in a porcine model, suggest that the approach of using microstirring pills to enhance drug bioavailability may hold promise toward obtaining similar improvements in humans.

## 3 Conclusion

We have reported on a novel microstirring pill platform technology that possesses built‐in mixing capability for in vivo oral drug delivery, leading to significantly enhanced drug absorption and bioavailability. We characterized the in vitro dissolution profiles at different microstirrer loadings and different fluid stirring speeds, demonstrating a substantially faster release of several model drugs at a speed that emulates gastric motility. By loading microstirring pills with tracer particles, we demonstrated the enhanced local fluid transport due to the pumping effect that was exerted. Microstirring pills, loaded with ASA, were orally administered to both mice and pigs. The acid‐driven propulsion and self‐stirring effect of the released microstirrers in the stomach environment led to greatly enhanced ASA uptake and bioavailability compared to static ASA pills. Since the encapsulated drugs and microstirrers were decoupled, the drug loading was not affected or compromised by the microstirrer excipient. These findings are of considerable relevance toward drug delivery in humans, particularly when considering the encouraging results obtained using the porcine model. In the future, the platform could be further adapted to enhance delivery to other regions of the GI tract, including the small intestines. Furthermore, the microstirrer pill technology could be applied to the delivery of a wide range of different therapeutic cargoes, such as peptides, proteins, or oral vaccines. On the other hand, other materials, such as Zn, Fe, Al (and its alloys), and CaCO_3_, could be utilized as microstirrers although special considerations must be applied as some of these materials have limited reactivity in intestinal biofluids and lower tolerance in the body compared to Mg.^[^ ^33^, ^34^, ^35^, ^36^, ^37^ ^]^

Overall, microstirring pills may help to bridge the microengine field with the pharmaceutical industry, and self‐stirring excipient could one day be used in state‐of‐the‐art pill formulations to modulate drug bioavailability upon oral administration. This simple yet elegant technology has a few unique strengths. First, it has a much higher translation potential: adding only an excipient material (synthetic microstirrers) into the pill formulation without changing anything else; second, it is a platform technology: agnostic to delivered drugs or pill formulations; last, it has tested validated in large animal model, representing the first time for microstirrers (or microengines) to be tested in a large animal model.

## 4 Experimental Section

##### Microstirrer Fabrication

Mg‐based microstirrers were prepared using commercial Mg microparticles (FMW20, TangShan Weihao Magnesium Powder Co.) with an average size of 20 ± 5 µm as the core. In order to remove impurities, the Mg microparticles were washed twice with acetone and dried under N_2_ current. Then, ≈10 mg of Mg microparticles were dispersed onto glass slides previously covered with 100 µL of 0.5% polyvinylpyrrolidone ethanolic solution (Spectrum Chemical MGF CORP). The Mg microparticles were then coated with TiO_2_ by atomic layer deposition (ALD) at 100 °C for 3000 cycles using a Beneq TFS 200 System. In this step, the exposed surface of the Mg particle was coated, leaving a small opening at the region where the Mg particles contacted the glass slide. Finally, the Mg microstirrers were retrieved by scratching them off the glass slide.

##### Microstirrer Characterization

Scanning electron microscopy (SEM) imaging of a Mg microstirrer was obtained with a FEI Quanta 250 ESEM instrument, using an acceleration voltage of 10 kV. Energy dispersive X‐ray analysis (EDX) mapping analysis was performed using an Oxford EDX detector attached to the SEM instrument and operated by Pathfinder software.

##### Pill Preparation

Microstirring pills were prepared by triturating and mixing lactose (Spectrum Chemical MGF Corp.) and maltose (Spectrum Chemical MGF Corp.) in a 60%/40% ratio. Once this mixture was homogeneous, microstirrers (0, 2, 5, or 10% of the total mixture weight) were incorporated and mixed in a mortar, and no changes in the microstirrer structure were observed during this mixing process. Model drugs were added at this step. Subsequently, an ethanol/water wetting solution (75%/25%) was added to the powder mixture to provide a paste‐like consistency. In some in vitro experiments, a yellow food dye was added at this stage to facilitate the visualization of the in vitro pill dissolution. Then, the paste was transferred to a cavity plate and each of the cavities was completely filled with the mixture by applying sufficient pressure to ensure tight packing. Immediately after filling the cavities, the cavity plate was lowered onto the peg plate until the wet pills were ejected. Finally, the microstirring pills were allowed to dry and harden over the peg plate at 65 °C for 2 h. Static pills were prepared following the same protocol with the exception of the addition of microstirrers.

##### Tracer Tracking and Analysis

Tracer tracking experiments were performed by adding 2 µm polystyrene (PS) tracer particles (9003‐53‐6, Polysciences Inc) to a solution of gastric acid stimulant with 0.6% Triton X‐100 surfactant (Sigma Aldrich) for the tracer only case (tracer particles were diluted ten times from a stock concentration of 2.62%). For the tracer trajectory analysis for the pill formulations, the same tracer particles were embedded into pills during the fabrication process at a 1.0% loading. Later, pills were dissolved in the same gastric acid simulant containing Triton X‐100. Videos were recorded at 30 fps on a Nikon Ti‐S/L100 inverted optical microscope coupled with a Hamamatsu digital camera C11440. Tracer tracking was performed with the NIS Elements AR 3.2 software. MSD calculation was performed with the publicly available MATLAB function (msdanalyzer) for a group of 40 particles (*n* = 40).^[^ ^38^ ^]^ Overlapped stacks of images were prepared with a publicly available ImageJ plugin, Flowtrace.^[^ ^39^ ^]^ The stacks correspond to 1 s of motion. The color of the images was inverted to show a black background. Lighter colored tails represent the most recent position of the tracer particle while darker colored tails represent the oldest position.

##### In Vitro ASA, L‐Dopa, and APAP Dissolution Analysis

Pills loaded with ASA (Spectrum Chemical MGF CORP), L‐Dopa (Sigma Aldrich), or APAP (Sigma Aldrich) were dissolved in 10 mL of gastric fluid simulant under stirring (200 rpm) at 37 °C. Aliquots of 25 µL were taken 0, 0.5, 1, 3, 5, 10, 15, and 30 min after the start of the experiment and analyzed for the corresponding drug, as well as after total pill dissolution. ASA was quantified using a salicylates ELISA kit (Neogen Corporation) following the manufacturer's specifications. L‐Dopa and APAP were quantified by using square‐wave voltammetry, measuring the anodic peak current corresponding to the oxidation of these drugs. These measurements involved a glassy carbon working electrode, Ag/AgCl reference electrode, and a Pt wire counter electrode, along with a CH660D potentiostat (CH instruments).

##### In Vivo ASA Delivery Study in Mice

To perform the in vivo ASA‐loaded microstirring pill delivery study in a murine model, male CD‐1 mice (Envigo Laboratories) were fasted overnight prior to the experiment. Then, mice (*n* = 6) were intragastrically administered with either ASA ‐loaded microstirring pills or static pills using a stainless‐steel X‐M dosing syringe (Torpac). A 50 µL blood sample was collected from the submandibular vein before administration and at 1, 5, 10, 30, and 60 min post‐administration. After spinning the blood for 5 min at 3000 × *g*, the serum was collected for quantifying the ASA concentration by ELISA.

##### In Vivo ASA Delivery Study in Pigs

To perform the in vivo ASA‐loaded microstirring pill delivery study in a porcine model, 3 months old female farm pigs (S&S Farms) 35 kg in weight were fasted overnight prior to the experiment. Then, pigs (*n* = 3) were anesthetized with ketamine, xylazine, and atropine, while monitoring their vital signs. Consequently, pigs were intragastrically administered with either ASA‐loaded microstirring pills or static pills using a flexible oral gavage tube. Blood samples were collected from the ear artery before administration and at 5, 15, 30, 60, 120, and 240 min post‐administration. After collecting the blood, it was left to clot at room temperature by leaving it undisturbed in a covered tube for 15–30 min; then, samples were centrifuged at 2000 × *g* for 5 min and the serum was collected for ASA quantification by ELISA.

##### Animal Care

Mice and pigs were housed in animal facilities at UC San Diego in compliance with local, state, federal, and National Institutes of Health guidelines. All the animal experiments were performed at an approved facility (AAALAC Accreditation Number 000503) according to protocols that were previously reviewed and approved by the Institutional Animal Care and Use Committee at UC San Diego.

##### Statistical Analysis

Data are presented as mean ± SD. In vitro studies: Statistical analysis of comparison of dissolution times of static and microstirring pills was performed using one‐way ANOVA (GraphPad Prism), ****p < 0.0001. Error bars represent standard deviation calculated from the dissolution of 3 different pills. Statistical analysis of velocity of tracer particles study was performed using one‐way ANOVA, ****p < 0.0001 (*n* = 40). In vivo studies in murine model: in the serum concentration of ASA after administration of static pills and microstirring pills study, error bars represent standard deviation calculated from the drug concentration in 6 different mice. In ASA AUC study for static and microstirring pills over 60 min, statistical analysis was performed using unpaired Student's *t*‐test, ****p < 0.0001, with *n* = 6. In vivo studies in porcine model: in the serum concentration of ASA after administration of ASA‐loaded microstirring pills and static pills statistical study, error bars represent standard deviation calculated from the drug concentration in 3 different pigs. In ASA AUC values for static and microstirring pills over 4 h study, statistical analysis was performed using unpaired Student's *t*‐test, *p < 0.05, with *n* = 3. No statistical methods were used to predetermine sample size. Studies were carried out in a non‐blinded fashion.

## Conflict of Interest

The authors declare no conflict of interest.

## Supporting information

Supporting Information

Supplemental Video 1

Supplemental Video 2

Supplemental Video 3

## Data Availability

Research data are not shared.
