# Supplementary material for: A Microstirring Pill Enhances Bioavailability of Orally Administered Drugs
Source: Adv Sci (Weinh). 2021 May 18;8(12):2100389. doi: 10.1002/advs.202100389 (PMC8224427; doi:10.1002/advs.202100389)
Supplement: Supplementary file 1 — Supporting Information [file ADVS-8-2100389-s002.pdf]

© 2021 Wiley-VCH GmbH

Supporting Information

for *Adv. Sci.,* DOI: 10.1002/advs.202100389

A Microstirring Pill Enhances Bioavailability of Orally Administered Drugs

Rodolfo Mundaca-Uribe[1], Emil Karshalev[1], Berta Esteban-Fernández de Ávila[1], Xiaoli Wei[1], Bryan Nguyen[1], Irene Litvan[2], Ronnie H. Fang[1], Liangfang Zhang,[1] and Joseph Wang.[1]**

Supporting Information

A microstirring pill enhances bioavailability of orally administered drugs

Rodolfo Mundaca-Uribe[1], Emil Karshalev[1], Berta Esteban-Fernández de Ávila[1], Xiaoli Wei[1], Bryan Nguyen[1], Irene Litvan[2], Ronnie H. Fang[1], Liangfang Zhang,[1]* and Joseph Wang.[1]*

[1]Department of Nanoengineering and Chemical Engineering Program, University of California San Diego, La Jolla, California 92093, United States.

[2]Department of Neurosciences, University of California San Diego, La Jolla, California 92093, United States.

*E-mail: josephwang@ucsd.edu; zhang@ucsd.edu

VIDEOS

Video S1. Dissolution of static and microstirring pills (prepared with 2%, 5%, and 10% of microstirrers, respectively) in 2 mL of 0.7M HCl solution.

Video S2. Dissolution of a static and a microstirring pill (10% micromotors), after 10 s in a 0.7 HCl solution stirred at 200 rpm.

Video S3. Fluid flow experiments with tracer particles.

SUPPORTING FIGURES

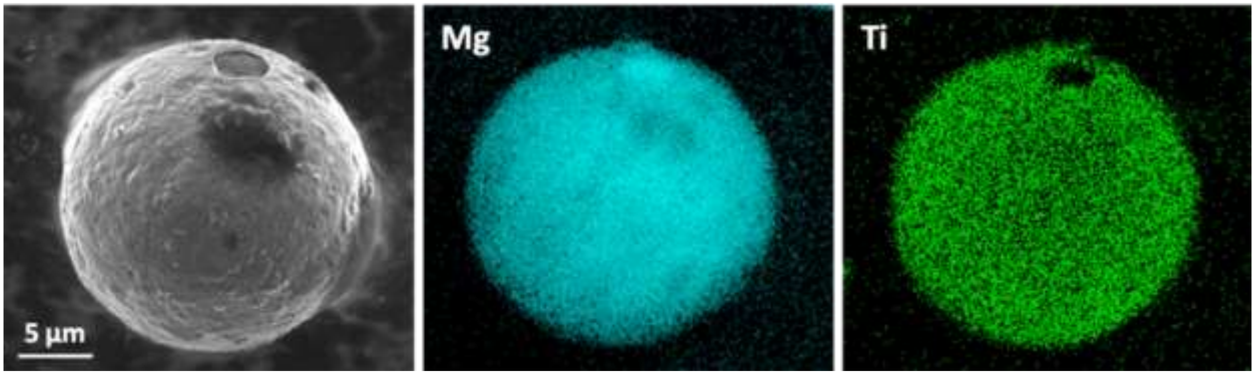

Figure S1. Scanning electron microscopy (SEM) images of a Mg-based microstirrer and energy-dispersive X-ray spectroscopy (EDX) images illustrating the distribution of elemental Mg (cyan) and Ti (green).

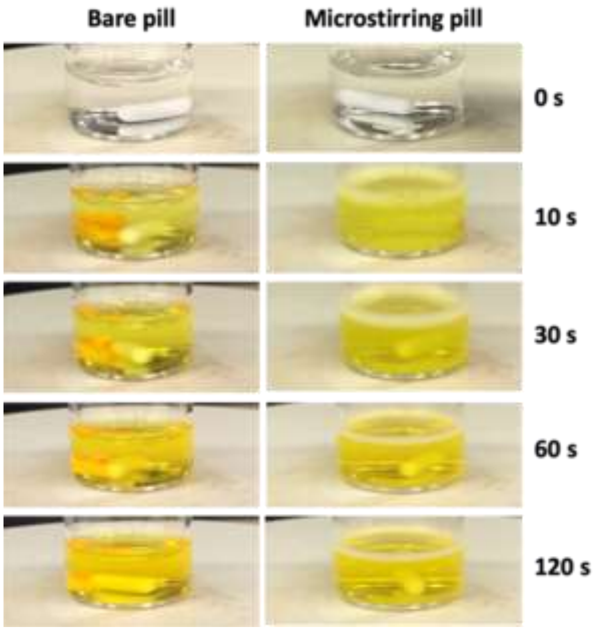

Figure S2. Time-lapse images showing the dissolution of a static pill and a microstirring pill (10% microstirrers) in 0.7 M HCl solution stirred at 200 rpm.

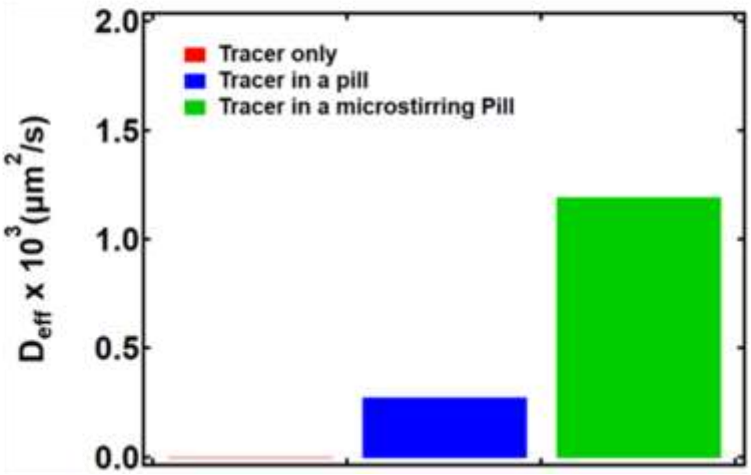

Figure S3. Diffusion coefficients of tracers only, tracers in a pill, and tracers in a microstirring pill in simulated gastric fluid.

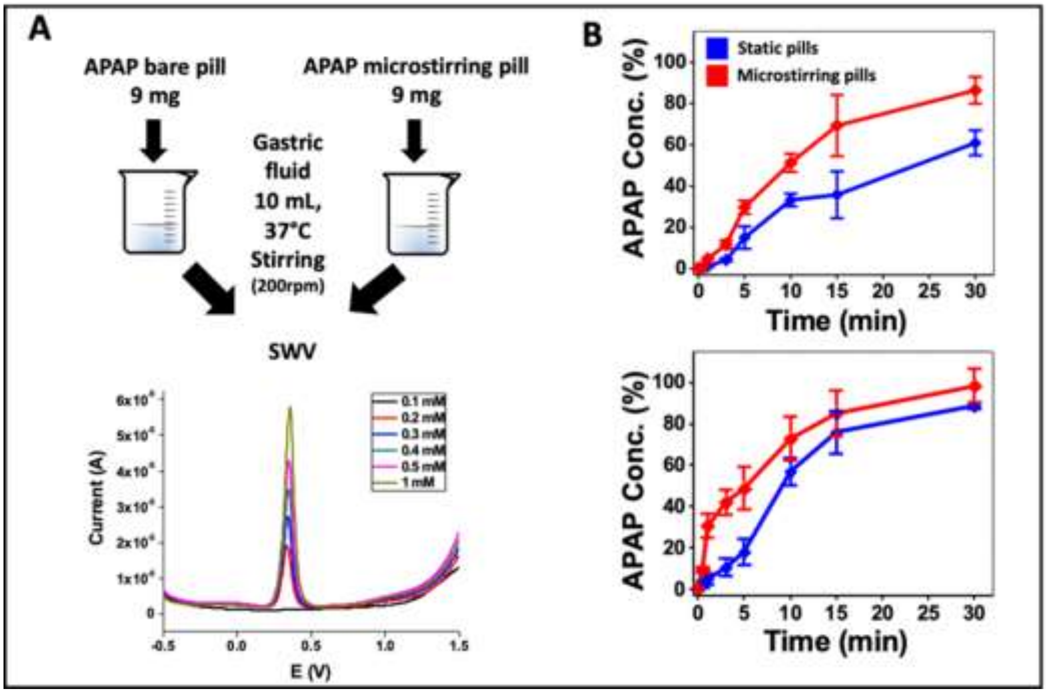

Figure S4. A) Schematic of the experimental design for quantifying acetaminophen (APAP) dissolution. **B)** Comparison of dissolution profiles of APAP between static and microstirring pills made with laboratory prepared excipients (top) and commercial excipients (bottom).

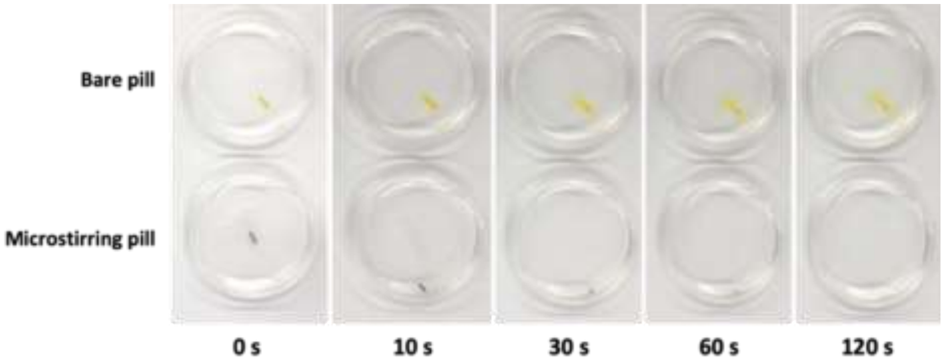

Figure S5. Time-lapse images showing the dissolution of a static pill and of a microstirring pill (16% microstirrers) in 2 mL of 0.7 M HCl solution. This formulation was used for the *in vivo* studies in mice.

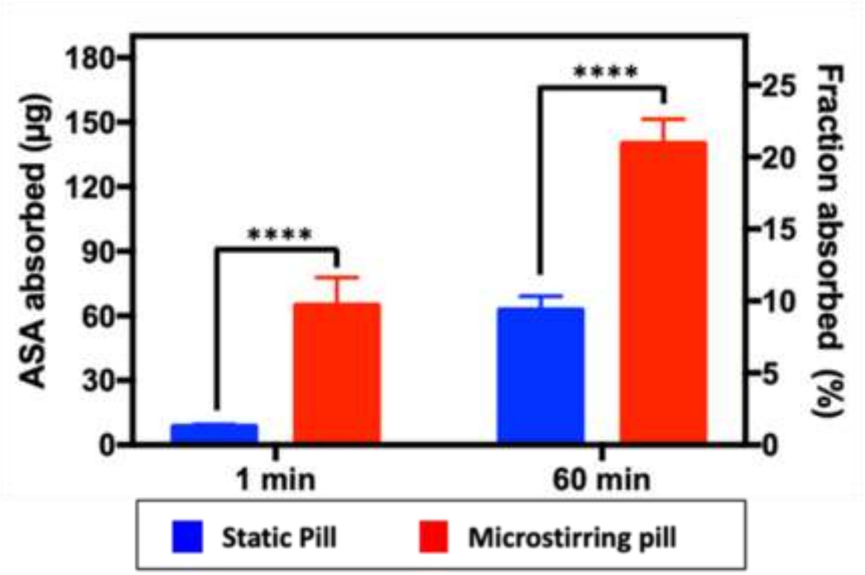

Figure S6. ASA absorbed fraction for static and microstirring pills at 1 and 60 min after administration.

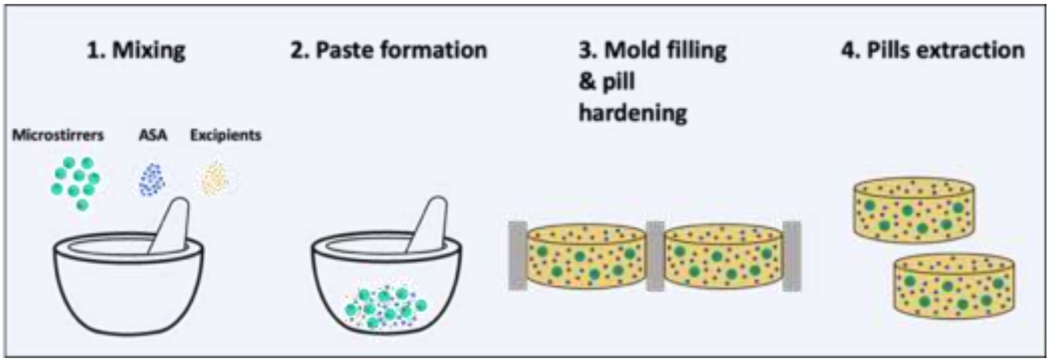

Figure S7. Schematic of the preparation of ASA-loaded microstirring pills.
